# Physical human-robot interaction of an active pelvis orthosis: toward ergonomic assessment of wearable robots

**DOI:** 10.1186/s12984-017-0237-y

**Published:** 2017-04-14

**Authors:** Nicolò d’Elia, Federica Vanetti, Marco Cempini, Guido Pasquini, Andrea Parri, Marco Rabuffetti, Maurizio Ferrarin, Raffaele Molino Lova, Nicola Vitiello

**Affiliations:** 1 The BioRobotics Institute, Scuola Superiore Sant’Anna, viale Rinaldo Piaggio, 34, 56025 Pontedera, Pisa Italy; 2 Fondazione Don Carlo Gnocchi IRCCS, Florence, Italy; 3 Fondazione Don Carlo Gnocchi IRCCS, Milan, Italy

**Keywords:** Wearable robotics, Active pelvis orthosis, Ergonomics, Passive degrees of freedom, Series-elastic actuation

## Abstract

**Background:**

In human-centered robotics, exoskeletons are becoming relevant for addressing needs in the healthcare and industrial domains. Owing to their close interaction with the user, the safety and ergonomics of these systems are critical design features that require systematic evaluation methodologies. Proper transfer of mechanical power requires optimal tuning of the kinematic coupling between the robotic and anatomical joint rotation axes. We present the methods and results of an experimental evaluation of the physical interaction with an active pelvis orthosis (APO). This device was designed to effectively assist in hip flexion-extension during locomotion with a minimum impact on the physiological human kinematics, owing to a set of passive degrees of freedom for self-alignment of the human and robotic hip flexion-extension axes.

**Methods:**

Five healthy volunteers walked on a treadmill at different speeds without and with the APO under different levels of assistance. The user-APO physical interaction was evaluated in terms of: (i) the deviation of human lower-limb joint kinematics when wearing the APO with respect to the physiological behavior (i.e., without the APO); (ii) relative displacements between the APO orthotic shells and the corresponding body segments; and (iii) the discrepancy between the kinematics of the APO and the wearer’s hip joints.

**Results:**

The results show: (i) negligible interference of the APO in human kinematics under all the experimented conditions; (ii) small (i.e., < 1 cm) relative displacements between the APO cuffs and the corresponding body segments (called stability); and (iii) significant increment in the human-robot kinematics discrepancy at the hip flexion-extension joint associated with speed and assistance level increase.

**Conclusions:**

APO mechanics and actuation have negligible interference in human locomotion. Human kinematics was not affected by the APO under all tested conditions. In addition, under all tested conditions, there was no relevant relative displacement between the orthotic cuffs and the corresponding anatomical segments. Hence, the physical human-robot coupling is reliable. These facts prove that the adopted mechanical design of passive degrees of freedom allows an effective human-robot kinematic coupling. We believe that this analysis may be useful for the definition of evaluation metrics for the ergonomics assessment of wearable robots.

## Background

In the field of human-centered robotics, exoskeletons are becoming relevant for addressing needs in the healthcare and industrial domains [1, 2], both as tools for rehabilitation treatment and clinical assessment [3, 4] and for augmented reality applications (haptics [5] or augmentation [6]). Despite the increasing interest and number of developed prototypes and commercial systems, the design of exoskeletons still has many open issues, such as those related to the development of the physical human-robot (HR) interface. Owing to their close interaction with the user, safety and ergonomics are critical features that heavily influence the functionality and the dependability of a wearable robot (WR) [7]. In general, these devices are designed to generate and transfer mechanical power to human joints: therefore, optimal kinematic coupling is required between the corresponding human and robot rotation axes [8].

Misalignment between the human and robot joint axes can cause undesired forces that overload human articulations, thus resulting in an uncomfortable or even painful interaction with the robot [9]. Undesired forces originating from joint axis misalignments (JAxM) can also lead the orthotic shells of the exoskeleton to slide along the human limb segments, leading to unreliable assistive torque transmission [10] and possible skin inflammation or even sores.

Unfortunately, the achievement of adequate human-robot joint axis alignment is not an easy condition to be fulfilled for two main reasons. First, it is not possible to know the exact location of the anatomical joint rotation axis without complex imaging techniques. Second, human articulations are not ideal rotational or spherical mechanical couplings; rather, they have more complex subject-dependent geometries that make the rotation axes fluctuate along the range of movement (ROM) [10].

As a consequence of the above considerations, most exoskeletons are provided with regulation mechanisms and/or passive degrees of freedom (pDoFs), in accordance with the guidelines proposed in [11]. In his work, Stienen and colleagues explained that it is possible to unload human articulations from undesired translational forces by decoupling joint rotations and translations by adding a certain number of passive DoFs to exoskeleton joints. Examples of WRs for both upper- and lower-limb assistance/rehabilitation equipped with passive DoFs have been reported in [11–14]. A more recent study also introduced a theoretical framework to identify the constructive parameters of the chain of passive DoFs that are necessary to cope with human flexion-extension articulations [7].

However, the introduction of passive DoFs into the design of a WR is not free of drawbacks; the tradeoff between the degrees of laxity [15] and the system complexity may affect the overall human-robot kinematics coupling [7]. On the one hand, by increasing the degree of laxity of the powered joints, there is a risk of increasing the overall inertia and friction of the moving parts. On the other hand, a lack of adequate laxity partially affects the human-robot joint axis self-alignment and thus hinders the spontaneous movement of the user. As a consequence, in the development and design of an exoskeleton, the assessment of its kinematic compatibility with user biomechanics is of paramount importance.

Many exoskeletons constitute the current state of the art; the variety of mechatronics designs, control systems, and human-machine interfaces are due to differences in the targeted users and expected usage. An extensive review of WRs, their design methodologies, and control strategies can be found in [16–18].

A category of powered WRs that is gaining an increasing level of attention is that of exoskeletons addressing the needs of people with *mild gait disturbances* (e.g., gait post-stroke hemiparesis, unilateral lower-limb amputation, senile gait, etc.), who may benefit from the use of light-weight assistive WRs to recover more stable, efficient, and independent locomotion [17, 19–21].

At The BioRobotics Institute (Scuola Superiore Sant’Anna, Pisa, Italy), we have recently developed a revised version of the active pelvis orthosis (APO) presented in [22], a wearable exoskeleton aimed at improving the gait energy efficiency of users affected by mild impairments through the assistance of hip flexion-extension (f/e) [23]. The main advancement of the new device over the previous version is the introduction of a chain of passive DoFs that allows the human f/e axis to align with that of the robot and simultaneously gives the user free hip abduction/adduction (a/a) and internal/external (i/e) rotations. The APO is interfaced with the wearer through tailored thermoplastic orthotic shells (namely, cuffs) to ensure maximum comfort.

The adopted design criterion is in line with the approaches proposed by several authors [7, 10, 11] for the development of exoskeletons that interact smoothly with the wearer. Nevertheless, to the best of our knowledge, no ergonomics evaluation methodology has been proposed in the literature and no clear definition of WR ergonomics has been given. Hereafter, we refer to ergonomics as the capability (of a WR) to smoothly interact with the user along the whole work space by “optimizing human well-being and overall system performance” [24] and without hindering natural kinematics or causing discomfort and/or injury.

The direct evaluation of ergonomics from ultimate determinants, such as comfort and risk of injury, may be performed only after long-term use. For this reason, the possibility to define the “level of ergonomics” from easily obtainable indirect measures that are related to ergonomics is attractive.

In this work, we carried out an experimental validation with healthy volunteers with the objective of assessing the quality of the user-APO physical interaction with particular reference to the chain of passive DoFs and the relative shifts between the APO frame and the human body at the physical interfacing areas. Using this specific device as an example, we discuss the specific design of a WR and propose a set of indicators that could be relevant for its evaluation in terms of ergonomics.

First, we analyzed the alteration of lower-limb joint kinematics by comparing the condition in which users walked without wearing the APO and all experimental conditions when they wore it. Human kinematics was recorded by means of an optoelectronic motion capture system. Secondly, we analyzed the stability of the physical interaction between the users and the APO by measuring: (i) the displacements between the APO orthotic cuffs and the wearer’s corresponding body segments, and (ii) the kinematic discrepancy between the APO and the wearer's hip f/e joint angle.

Besides the ease of measurement, the following hypotheses form the rationale behind the choice of these variables: (i) deviation from natural kinematics is recognized as a negative effect on the wearer; (ii) relative displacements at the HR interface may cause skin irritations or sores and therefore discomfort or injuries; (iii) HR kinematic discrepancy together with relative displacements at the interface may reveal possible JAxM, which are the cause of residual forces onto articulations and possibly pain or injuries after prolonged use.

We carried out this study being aware that: (i) optoelectronic systems have been widely used to measure human gait kinematics [25], also during orthosis-assisted locomotion [26, 27], and (ii) the feasibility of measuring displacements in the order of millimeters (in the range 3.2–6.7 mm) using a video-based motion capture system, such as that used in the present application, has been already demonstrated [28, 29].

## Methods

### Participants

Five healthy adults (74.4 ± 6.8 kg, 1.73 ± 0.07 m, 29.2 ± 6.3 years old) were enrolled for the study. All participants signed an informed consent before starting the experimental sessions. The research procedures were conducted at the premises of Fondazione Don Carlo Gnocchi (Firenze, Italy) in accordance with the Declaration of Helsinki, after the approval of the local Ethical Committee.

### Active pelvis orthosis

The APO is a bilateral powered exoskeleton, and it is constituted of three main subsystems: the mechanical structure, the actuation units, and the control system. In the following, we provide a description that summarizes its main features.

**Fig. 1:**
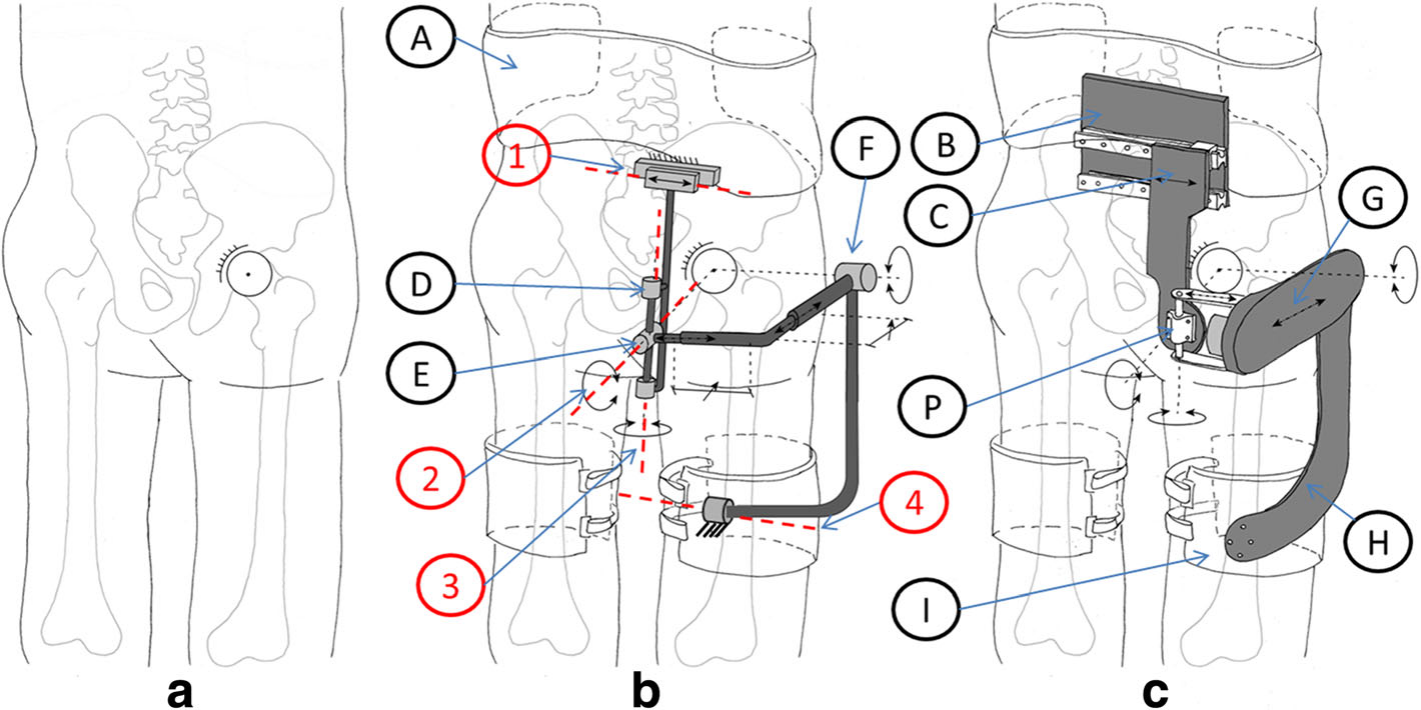
**a** Human skeleton covered by soft tissues. **b** Human body with wearable robotic chain schematic: (1) passive translational DoF, (2) abduction/adduction rotation passive DoF, (3) internal/external rotation passive DoF, (4) rotation passive DoF, (**a**) pelvis cuff, (**d**) internal/external rotation joint, (**e**) abduction/adduction joint, (**f**) flexion/extension joint. **c** Human body coupled with exoskeleton: **b** carbon-fiber plate, **c** sliding carbon-fiber plate, **g** lateral extensible arm, **h** thigh linkage, **i** thigh cuff. For the sake of clarity, only the right part of the bilateral APO is represented

The mechanical structure of the APO is symmetrical with respect to the sagittal plane (Fig. 1). Each side of the robot is composed of two main subsystems, namely, the chain of passive DoFs and the transmission means that transfer the assistive torque from the actuation unit to the human hip articulation.

Each chain of passive DoFs originates from a main posterior carbon fiber plate (i.e., the frame of the chain of passive DoFs), which connects the exoskeleton to the wearer’s trunk by means of an orthotic cuff. Another carbon-fiber plate can slide horizontally against the frame by means of a passive translational DoF (axis number 1 in Fig. 1). The sliding plate houses two passive rotational DoFs, whose axes of rotation (namely, axes number 2 and 3 in Fig. 1) are orthogonal and cross each other at point P. Thanks to the combined action of the translational passive DoF, the rotation axis number 2 can be aligned with the human hip a/a axis. The range of motion of axis number 2, and, consequently, that of the user’s a/a, is restricted to −15° to +20° via mechanical end stops. The concomitant movement of the translational passive DoF and that of axis number 3 allow the user to also have a free hip i/e. The ROM of axis number 3 is restricted to –10° to +10° by mechanical end stops. This kinematic chain of passive DoFs connects the main carbon fiber plate to a lateral arm, also made of carbon fiber. The distance between the left and right lateral arms on the frontal plane can be manually adjusted to fit the width of the wearer’s pelvis. Each lateral arm is made of two telescopic shells that can slide against each other in order to maximally align the human and robot f/e axes in the sagittal plane by manually tuning their lengths. A thigh linkage rotates around the f/e axis and couples with the wearer’s thigh via an orthotic shell. Finally, an additional rotational DoF is inserted between each link and the cuff (axis number 4 in Fig. 1); this allows the alignment of the cuff and thigh longitudinal axes, thus providing considerable stability during movement. The APO kinematic chain design is patent pending [30].

The transmission system connects the actuation unit placed on the rear part of the lateral arm to a driven pulley placed coaxially with the hip f/e axis by means of a steel cable (U8191517, Carl Stahl®GmbH, Suessen, Germany) in a capstan configuration. The stiffness of the cable is 250 N/mm, equivalent to a torsional stiffness at the hip joint of 756 Nm/rad. The rotation axis of the actuation unit is parallel to the hip f/e axis.

Each actuation unit is a series elastic actuator (SEA). Each SEA is composed of a 70 W DC motor (EC60, Maxon Motor®, Sachseln, Switzerland), a 100:1 harmonic drive (CPL-14A-100-2A, Harmonic Drive®, Limburg, Germany), and a custom torsional spring (patent pending) having a stiffness of 100 Nm/rad. Two absolute 17-bit Rotary Electric Encoder™ units (DS-37 and DS-25 Netzer Precision Motion Sensors Ltd, Misgav, Israel) measure the spring deformation and the actual hip joint angle, respectively.

The control system has a hierarchical structure made of a low- and a high-level layer. For the control system, we adopted the same control architecture as that described in [22]; hereafter, its main features are recapped for the sake of clarity. The low-level layer is a closed-loop torque control. The controller is a 2-pole-2-zero compensator. The closed-loop compensator allows for a relatively high closed-loop bandwidth (namely, 15.5 Hz) and low joint residual parasitic stiffness (lower than 1 Nm/rad in the typical frequency range of walking). The high-level assistive control aims at computing a desired assistive torque profile during the stride for each of the two powered hip joints. It is based on the model-free algorithm presented in [31]. It relies on adaptive oscillators, mathematical tools [32] that—when coupled with a kernel-based non-linear filter—can track, estimate, and predict quasi-periodic signals (e.g., hip angles during gait) with zero-delay. Hence, during ground-level walking tasks, it is possible to determine the phase φ, frequency, and envelope of each hip joint angle and to reliably predict the joint angle during the stride period thanks to an adjustable phase shift Δφ (Δφ = 0.628 rad in this work). The assistive reference torque is provided by: ${\uptau}_{\mathrm{des}}={\mathrm{K}}_{\mathrm{v}}\cdot \left[{\widehat{\uptheta}}_{\mathrm{j}}\left(\upvarphi +\Delta \upvarphi \right)-{\widehat{\uptheta}}_{\mathrm{j}}\left(\upvarphi \right)\right]$, where K_v_ [Nm/rad] is an adjustable virtual stiffness and $\widehat{\uptheta_{\mathrm{j}}}\left(\upvarphi \right)$ and $\widehat{\uptheta_{\mathrm{j}}}\left(\upvarphi +\Delta \upvarphi \right)$ are the hip joint angle estimate and its predicted future value, respectively. This means that thanks to the virtual stiffness, we can attract the hip f/e angle from the current position ${\widehat{\uptheta}}_{\mathrm{j}}\left(\upvarphi \right)$ to the future ${\widehat{\uptheta}}_{\mathrm{j}}\left(\upvarphi +\Delta \upvarphi \right)$ as a result of the application of a torque τ_des_. In this experiment, the parameters of the assistive controller were set according to [31].

### Experimental protocol

All volunteers walked barefoot on a treadmill at three different speeds (slow, normal, and fast, named V1, V2, and V3, respectively) and under five modalities, namely: (i) without wearing the APO mechanics, but with the pelvis orthotic cuff (natural walking, NW); (ii) wearing the APO in the zero-torque control mode (transparent mode, TM); (iii)–(v) assistive mode with three different levels of assistance (low, moderate, and high assistive modes, named AM1, AM2, and AM3, respectively). The velocity V2 was selected according to the principle of dynamic similarities [33] and was thus calculated as $\mathrm{V}2=\sqrt{{\mathrm{F}}_{\mathrm{r}}\cdot \mathrm{g}\cdot \mathrm{L}}$, where F_r_ is the Froude number, g is the gravity constant, and L is the leg length (measured from the greater trochanter prominence to the lateral malleolus). In this experiment, F_r_ = 0.1. V1 and V3 were selected to be equal to V2 ± 0.25 V2. Each subject walked in all conditions (in the order: NW, TM, AM1, AM2, and AM3) at all different speeds (in the order: V1, V2, and V3). Each trial consisted of 20 strides.

The desired assistance level was set according to the following methodology. During a familiarization session, each volunteer was requested to walk at V3 while the experimenter progressively increased the value of K_v_. The value of K_v_ for AM3 was that corresponding to the highest level of assistance that the subject considered as comfortable. In fact, all subjects reported discomfort for high values of K_v_. When the peak torque—normalized to body weight—exceeded an across-subjects average value of 0.14 Nm/kg, the human and robotic hip joint kinematics difference increased, thus resulting in an assistive action that is not compliant with human biomechanics. We will further discuss this issue in the discussion session. Once the K_v_ upper limit was identified, it was scaled down by 33 and 66% for the AM2 and AM1 conditions, respectively.

Owing to the APO modular architecture, it was possible to wear only the pelvis cuff in NW. In all other assisted conditions, including TM, thigh cuffs were also worn.

### Data acquisition and processing

**Fig. 2:**
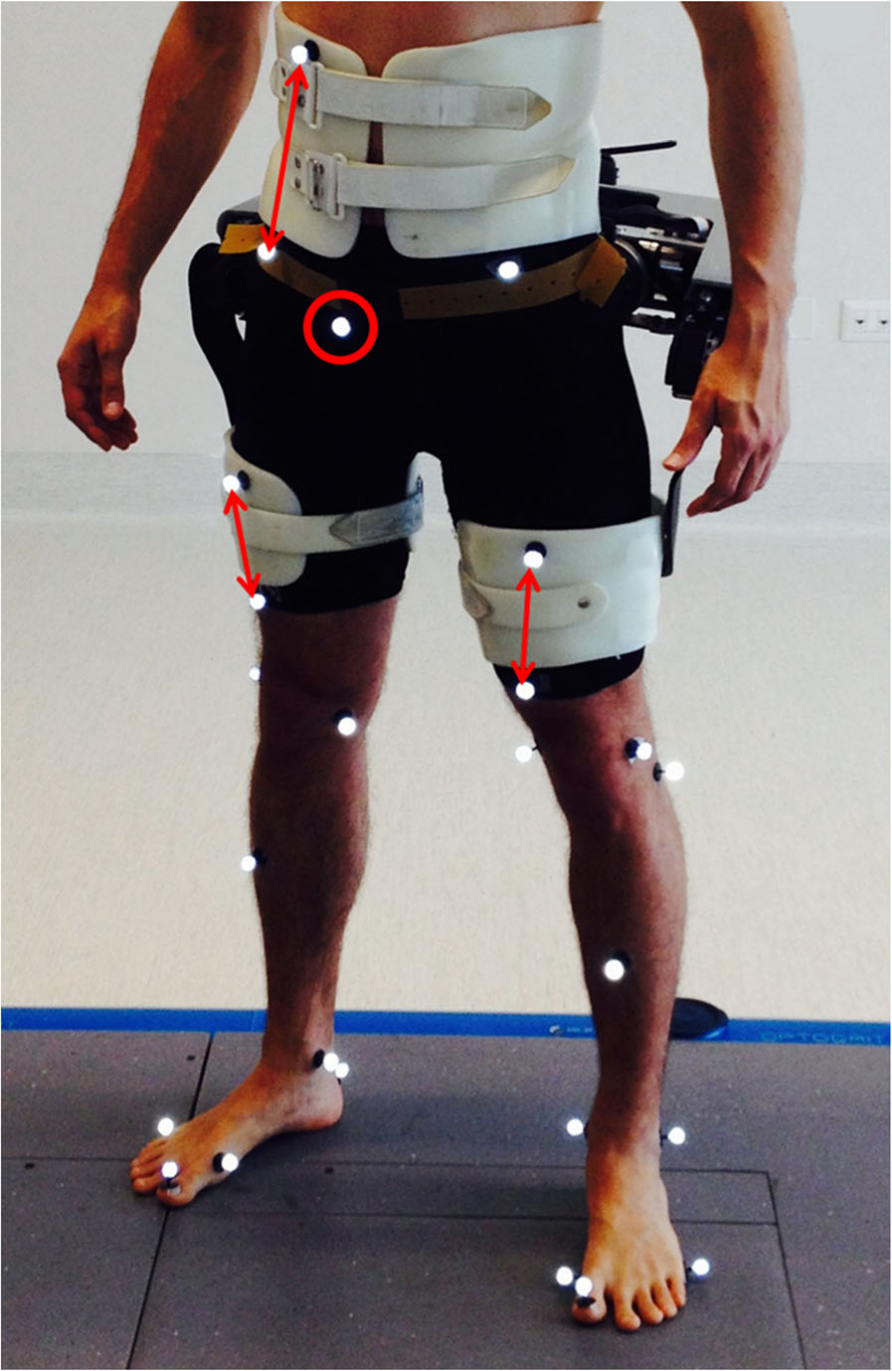
Adapted LAMB model marker setup. Posterior superior iliac spine landmark, hidden by the exoskeleton, was reconstructed through a marker placed on top of a pelvis-anchored stick (encircled in *red*). *Red arrows* shows which marker are used to calculate body-cuff relative displacements

The APO actual hip joint angle and torque were measured by using the information from the encoders. Lower-limb kinematics and the movement between the orthotic cuffs and the corresponding body segments were measured by means of an optoelectronic system (SmartD, BTS, Milan, Italy) detecting spherical passive markers placed on specific points on the robot and user. Standard software (Smart Tracker, BTS, Milan, Italy) was used to compute the 3D coordinates of the markers. Ad-hoc post-processing was performed in the MATLAB environment (MathWorks, Natick, MA, USA). Human kinematics was calculated according to an adapted LAMB model [34] (see Fig. 2), in which the required posterior superior iliac spine landmark, hidden by the exoskeleton, was reconstructed through a marker placed on top of a pelvis-anchored stick. All acquired data were segmented according to heel strikes and resampled from 0 to 100% of the stride cycle. The heel strikes were detected with a dedicated algorithm that processes the 3D coordinates of feet markers.

From the collected data, we computed the following variables, which provided a quantitative assessment of the level of ergonomics: (i) the root mean square (RMS) of the difference of the hip, knee, and ankle f/e angle—measured by the motion capture system—between the NW and TM/AM conditions; we named these variables ‘human hip angle deviation’ (H-HAD), ‘human knee angle deviation’ (H-KAD), and ‘human ankle angle deviation’ (H-AAD), respectively; (ii) the RMS of the difference between the APO hip f/e angle, recorded by the joint encoder, and the anatomical hip f/e angle, computed through the motion capture system; we named this variable ‘human-robot hip angle deviation’ (HR-HAD); (iii) the standard deviation (SD) of the relative displacements between the markers placed on the orthotic cuffs of the APO and those placed on their corresponding body segments; we named this set of variables ‘physical human-robot interface displacement’ (pHR-ID); (iv) the human joints’ ROM in the sagittal plane (hip, knee, and ankle) and in the frontal plane (only at the hip); and (v) spatio-temporal parameters (i.e., step length, stance time, and cadence).

In the case of the pHR-ID calculation, we used the SD instead of the RMS because it is more adequate for measuring the relative displacements of two points in space, without considering the constant offset between them.

The reference value used to evaluate H-HAD, H-KAD, and H-AAD in all walking conditions was the average intra-subject variability, defined as the among-subjects average of intra-subject variability. The intra-subject variability, calculated in each walking condition for one subject, is here defined as the average SD in a stride period. Hence, the intra-subject variability quantifies the natural differences of the f/e angles for one subject, while the average intra-subject variability captures the mean of these natural kinematic differences considering all subjects.

The relative displacement between markers placed on the same rigid body, namely, the pelvis cuff of the APO during walking, averaged over all conditions, was taken as the noise level of the experimental setup (2.3 ± 0.8 mm).

Since we verified that no significant left/right differences (*p* < 0.05) were present in all data, in the following, only the right-side data are presented.

In order to evaluate significant differences due to the effects of speed and the walking modality, a two-way ANOVA (*p* < 0.05) with the Fisher LSD post-hoc comparison was performed.

## Results

All volunteers completed the experimental tests successfully and wore the APO in the TM and AMs without reporting discomfort. All subjects showed an average pelvis anteversion of 10 ± 1° when wearing the APO with respect to not wearing it (in accordance with [35]).

### Spatio-temporal parameters

Table 1 reports the spatio-temporal parameters for different trials. When considering speeds, the stance time shows a negative trend associated with speed increase, while cadence shows a positive trend; conversely, none of the spatio-temporal parameters show any trend associated with the assistance level. All spatio-temporal parameters, except step length, show significant differences due to speed increase. When comparing natural walking with the TM/AMs, all spatio-temporal parameters except the stance time show no significant differences. In particular, the average stance time in AM2 is slightly larger than in NW. When comparing walking conditions in which the exoskeleton was worn, no statistically relevant differences are found, except for the stance time, which shows a difference between TM and AM2.

**Table 1:** Mean ± SD of spatio-temporal parameters categorized by speed and walking modality

	Step length [% of stride length]			Stance time [% of gait cycle]			Cadence [Steps/min]		
Walking condition	V1	V2	V3	V1	V2	V3	V1	V2	V3
NW	51 ± 1	50 ± 1	50 ± 1	71 ± 1	68 ± 1	66 ± 1	78 ± 9	94 ± 8	109 ± 8
TM	50 ± 2	50 ± 1	50 ± 1	71 ± 1	68 ± 1	66 ± 1	79 ± 7	94 ± 7	106 ± 8
AM1	50 ± 1	50 ± 1	50 ± 1	72 ± 1	68 ± 1	66 ± 1	78 ± 12	93 ± 9	107 ± 9
AM2	50 ± 1	50 ± 1	50 ± 1	72 ± 1	69 ± 1	67 ± 0	80 ± 13	94 ± 11	108 ± 9
AM3	49 ± 2	50 ± 1	50 ± 1	71 ± 1	69 ± 1	67 ± 1	80 ± 13	95 ± 10	108 ± 9

Condition coding: slow speed (V1), self-selected speed (V2), fast speed (V3), natural walking –no APO- (NW), transparent mode -APO shadows the wearer- (TM), low assistance (AM1), moderate assistance (AM2), high assistance (AM3)

### Kinematics

**Fig. 3:**
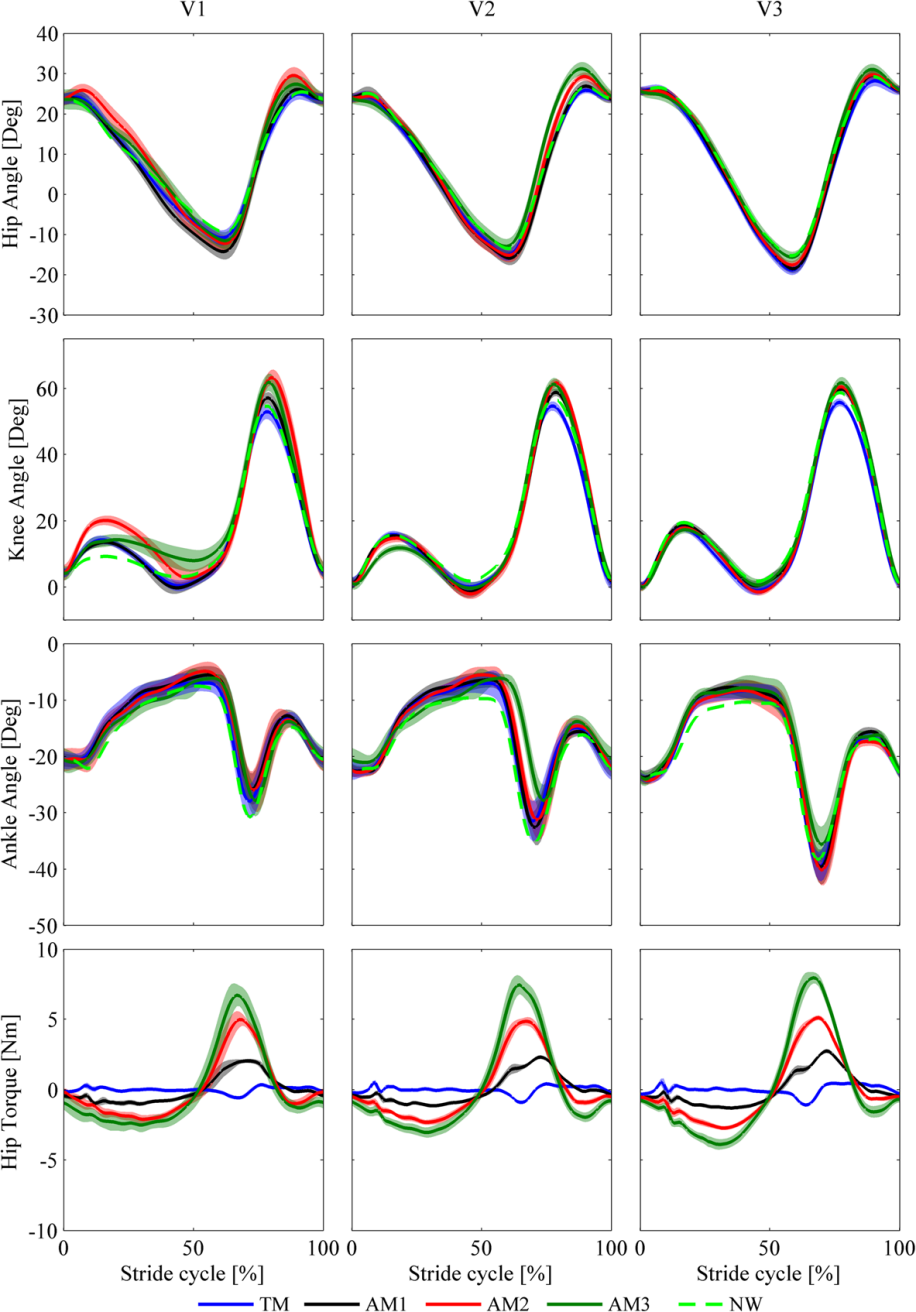
Kinematics and kinetics for all speeds (slow (V1), self-selected (V2), fast (V3)) and walking conditions (natural walking –no APO– (NW, *light green dashed lines*), transparent mode –APO shadows the wearer- (TM, *blue lines*), low assistance (AM1, *black lines*), moderate assistance (AM2, *red lines*), high assistance (AM3, *dark green lines*)). Each line represents the stride average and one SD band for each trial from one representative subject

In Fig. 3, we present the kinematics of the hip, knee, and ankle in the sagittal plane and the kinetics of the hip for one representative subject.

**Fig. 4:**
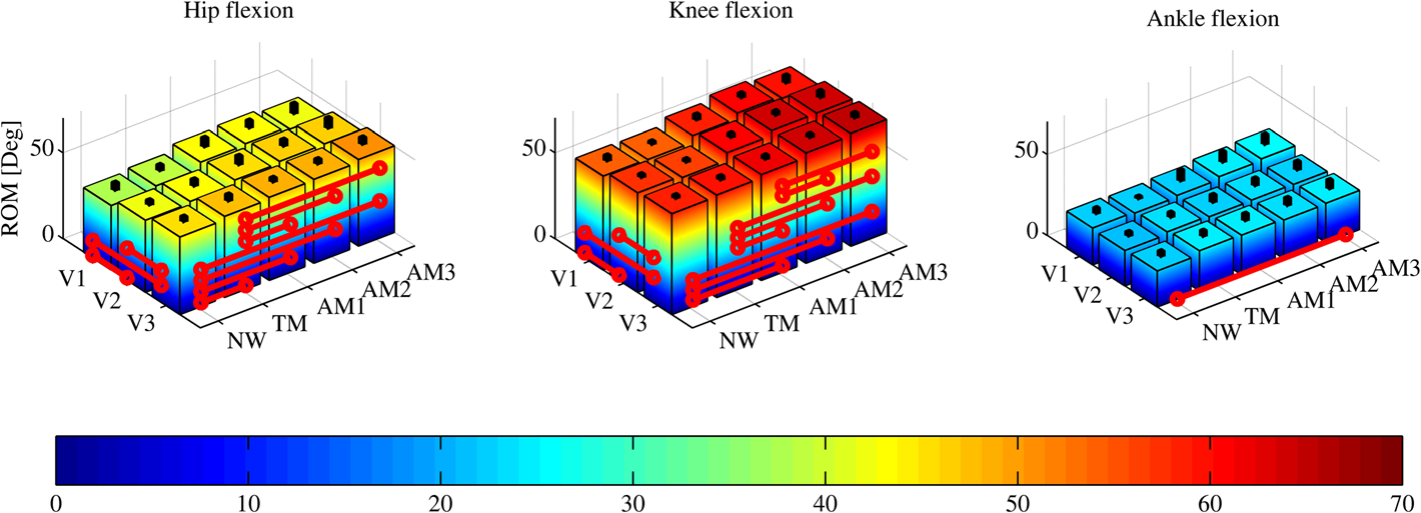
Hip, knee and ankle flexion-extension angle range of motion (ROM) for all speeds (slow (V1), self-selected (V2), fast (V3)) and walking conditions (natural walking –no APO- (NW), transparent mode -APO shadows the wearer- (TM), low assistance (AM1), moderate assistance (AM2), high assistance (AM3)). Each colored column is the average ROM; *black columns* represent one SD band. *Red lines* show significant (two-way ANOVA (*p* < 0.05) with Fisher LSD post-hoc) differences

The hip and knee ROMs (see Fig. 4 and Table 2) show a positive trend associated with assistance and speed increase. With respect to NW, the hip ROM significantly increases when wearing the exoskeleton in the TM and AMs, while the knee ROM significantly increases in the AMs but not in the TM; the ankle ROMs do not show any significant difference, except for AM3. When comparing the AMs, we notice no significant differences in the ROMs for the hip and ankle, while the knee ROM in AM1 is significantly lower than those in AM2 and AM3.

**Table 2:** Mean ± SD of ROM categorized by speed and walking modality

	Hip [Deg]			Knee [Deg]			Ankle [Deg]		
Condition	V1	V2	V3	V1	V2	V3	V1	V2	V3
NW	37 ± 4	42 ± 3	45 ± 3	55 ± 3	58 ± 2	59 ± 2	23 ± 4	22 ± 3	22 ± 3
TM	39 ± 3	43 ± 4	47 ± 4	54 ± 2	57 ± 2	60 ± 2	22 ± 1	23 ± 3	25 ± 4
AM1	43 ± 5	45 ± 5	48 ± 2	58 ± 3	61 ± 2	62 ± 2	23 ± 7	23 ± 5	24 ± 5
AM2	44 ± 4	46 ± 4	49 ± 3	61 ± 3	64 ± 2	64 ± 2	25 ± 7	23 ± 5	24 ± 5
AM3	42 ± 6	46 ± 6	50 ± 4	60 ± 4	64 ± 4	65 ± 3	26 ± 6	23 ± 5	24 ± 6

Condition coding: slow speed (V1), self-selected speed (V2), fast speed (V3), natural walking –no APO- (NW), transparent mode -APO shadows the wearer- (TM), low assistance (AM1), moderate assistance (AM2), high assistance (AM3)

**Fig. 5:**
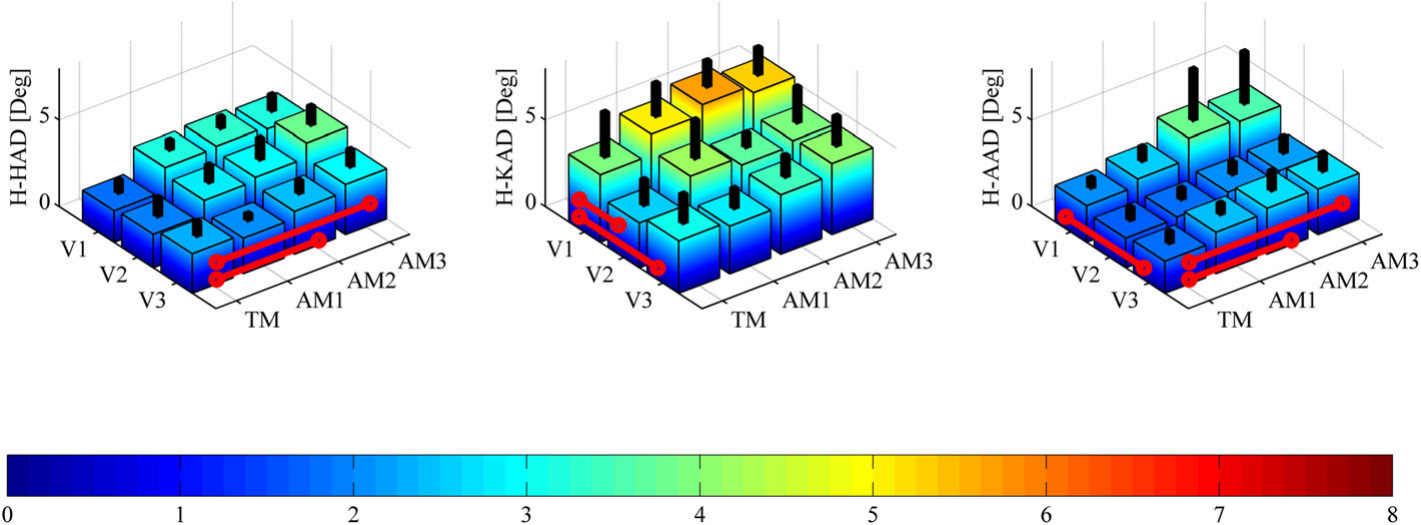
Mean ± SD of human hip angle deviation (H-HAD, left), human knee angle deviation (H-KAD, center) and human ankle angle deviation (H-AAD, right) for all speeds (slow (V1), self-selected (V2), fast (V3)) and walking conditions (natural walking –no APO- (NW), transparent mode -APO shadows the wearer- (TM), low assistance (AM1), moderate assistance (AM2), high assistance (AM3)). *Black columns* represent one SD band. *Red lines* show significant (a two-way ANOVA (*p* < 0.05) with Fisher LSD post-hoc) differences

**Fig. 6:**
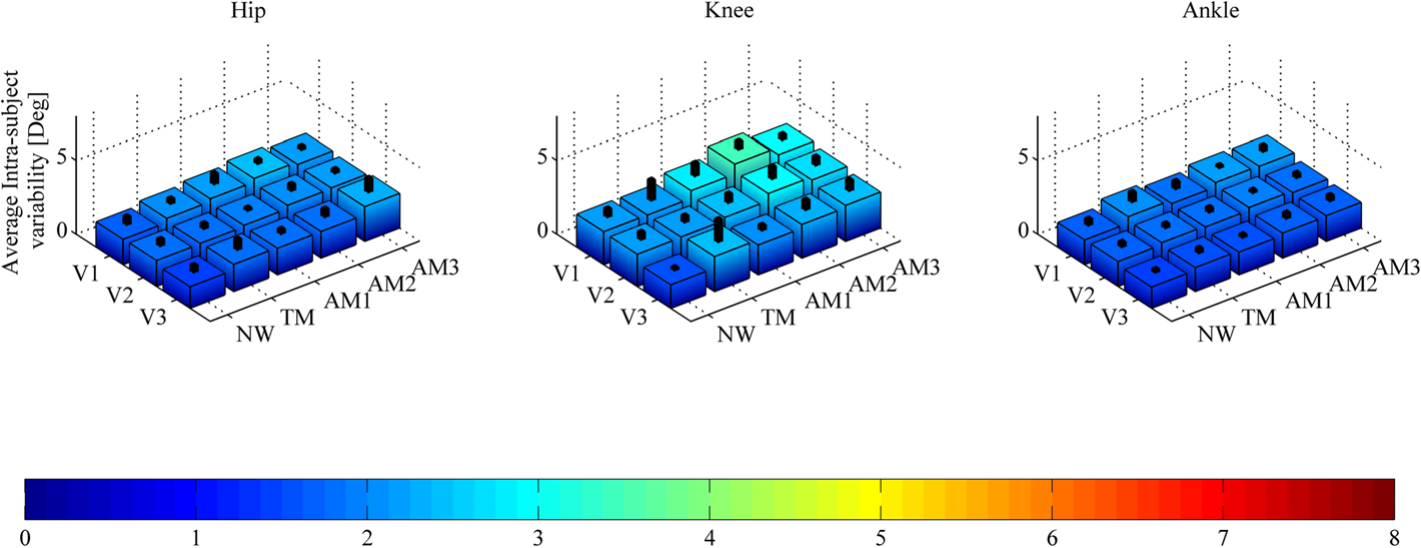
Average intra-subject variability of hip (left), knee (*center*), ankle (*right*) flexion-extension angle for all speeds (slow (V1), self-selected (V2), fast (V3)) and walking conditions (natural walking –no APO- (NW), transparent mode -APO shadows the wearer- (TM), low assistance (AM1), moderate assistance (AM2), high assistance (AM3)). *Black columns* represent one SD band

Despite the hip and knee ROM increase with assistance, the overall f/e angle trajectories in all walking conditions are highly overlapped, showing consistency between the kinematics in NW and in other walking conditions. In fact, when considering H-HAD, H-KAD, and H-AAD (see Fig. 5 and Table 3), which account for the global difference between the kinematics in NW and in other conditions, we obtain values comparable with the average intra-subject variability of the hip, knee, and ankle f/e angles (see Fig. 6 and Table 4). H-HAD ranges from 1.8 ± 0.8° in V1-TM to 3.9 ± 1.1° in V2-AM3, while the average intra-subject variability of the hip f/e angle ranges from 1.5 ± 0.5° in V3-NW to 2.4 ± 1.0° in V3-AM3. H-KAD ranges from 2.6 ± 1.5° in V2-TM to 5.7 ± 1.5° in V1-AM2, while the average intra-subject variability of the knee f/e angle ranges from 1.6 ± 0.2° in V3-NW to 3.4 ± 0.6 in V1-AM2. H-AAD ranges from 1.7 ± 0.8° in V2-TM to 3.8 ± 3.0° in V1-AM2, while the average intra-subject variability of the ankle f/e angle ranges from 1.4 ± 0.2° in V3-NW to 2.2 ± 0.2 in V1-AM2.

**Table 3:** Mean ± SD of human hip angle deviation (H-HAD), human knee angle deviation (H-KAD) and human ankle angle deviation (H-AAD) categorized by speed and walking modality

	H-HAD [Deg]			H-KAD [Deg]			H-AAD [Deg]		
Condition	V1	V2	V3	V1	V2	V3	V1	V2	V3
TM	1.8 ± 0.8	2.0 ± 1.1	2.9 ± 0.9	3.9 ± 2.6	2.6 ± 1.5	3.0 ± 1.7	2.1 ± 0.6	1.7 ± 0.8	1.9 ± 0.7
AM1	3.2 ± 0.6	2.8 ± 1.1	2.1 ± 0.4	5.1 ± 1.9	4.2 ± 2.2	2.8 ± 1.2	2.5 ± 0.9	1.8 ± 0.7	2.4 ± 0.7
AM2	3.3 ± 0.7	3.0 ± 1.3	2.5 ± 0.8	5.7 ± 1.5	3.7 ± 0.9	3.5 ± 1.3	3.8 ± 3.0	2.1 ± 0.8	2.7 ± 1.2
AM3	3.2 ± 1.0	3.9 ± 1.1	2.9 ± 1.0	5.3 ± 1.6	4.0 ± 2.1	4.1 ± 1.7	3.6 ± 3.0	2.2 ± 1.0	2.6 ± 1.1

Condition coding: slow speed (V1), self-selected speed (V2), fast speed (V3), transparent mode -APO shadows the wearer- (TM), low assistance (AM1), moderate assistance (AM2), high assistance (AM3)

**Table 4:** Mean ± SD of average intra-subject variability of hip, knee and ankle flexion-extension angle categorized by speed and walking modality

	Hip [Deg]			Knee [Deg]			Ankle [Deg]		
Condition	V1	V2	V3	V1	V2	V3	V1	V2	V3
NW	1.7 ± 0.6	1.8 ± 0.5	1.5 ± 0.5	2.2 ± 0.6	2.2 ± 0.5	1.6 ± 0.2	1.7 ± 0.4	1.7 ± 0.3	1.4 ± 0.2
TM	2.0 ± 0.4	1.8 ± 0.4	1.8 ± 0.7	2.2 ± 1.4	2.2 ± 0.5	2.4 ± 1.6	2.1 ± 0.7	1.8 ± 0.3	1.6 ± 0.3
AM1	2.2 ± 0.7	1.9 ± 0.2	1.8 ± 0.3	2.8 ± 0.9	2.4 ± 0.6	2.0 ± 0.4	1.9 ± 0.5	1.8 ± 0.3	1.5 ± 0.2
AM2	2.4 ± 0.3	2.1 ± 0.5	1.8 ± 0.6	3.4 ± 0.6	2.9 ± 0.9	2.3 ± 0.7	2.2 ± 0.2	2.0 ± 0.2	1.7 ± 0.4
AM3	2.2 ± 0.3	2.1 ± 0.3	2.4 ± 1.0	2.9 ± 0.4	2.7 ± 0.6	2.4 ± 0.7	2.1 ± 0.4	1.8 ± 0.4	1.7 ± 0.4

Condition coding: slow speed (V1), self-selected speed (V2), fast speed (V3), natural walking –no APO- (NW), transparent mode -APO shadows the wearer- (TM), low assistance (AM1), moderate assistance (AM2), high assistance (AM3)

When comparing the AMs, H-HAD and H-KAD show no statistically significant differences related to the assistance level increase. Conversely, H-HAD and H-AAD show significant differences when considering TM vs AM2/AM3.

H-HAD is not significantly affected by speed increments, while H-KAD in V1 shows significant differences from V2 and V3; H-AAD in V1 is significantly different from V3.

### Physical human-robot interface displacements

**Fig. 7:**
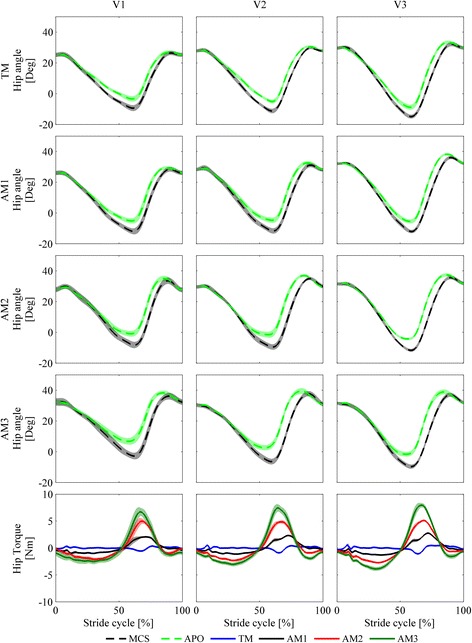
Physical human-robot interface displacement pHR-ID (mean ± SD) for pelvis cuff (*left*) and right thigh cuff (*right*) for all speeds (slow (V1), self-selected (V2), fast (V3)) and walking conditions (transparent mode -APO shadows the wearer- (TM), low assistance (AM1), moderate assistance (AM2), high assistance (AM3)). *Black columns* represent one SD band. *Red lines* show significant (a two-way ANOVA (*p* < 0.05) with Fisher LSD post-hoc) differences

Table 5 and Fig. 7 report the pHR-ID data for all trials and cuffs. When considering the walking modalities, we notice a positive trend in the displacements between the cuffs and their corresponding body segments that is related to the increment of delivered assistance. When considering speeds, only thigh cuffs show a positive trend associated with speed increase. The natural walking condition is only reported for the pelvis, since thigh cuffs could not be worn without the APO being donned.

**Table 5:** Mean ± SD of pHR-ID categorized by speed and walking modality

	Pelvis [mm]			Right thigh [mm]		
Condition	V1	V2	V3	V1	V2	V3
NW	2.4 ± 1.2	2.1 ± 0.9	2.2 ± 0.8	-	-	-
TM	2.2 ± 0.8	2.0 ± 0.6	2.4 ± 0.8	4.0 ± 0.6	4.4 ± 0.7	5.1 ± 1.0
AM1	2.2 ± 0.8	3.1 ± 2.2	2.6 ± 1.3	5.1 ± 1.7	5.5 ± 1.5	6.1 ± 1.9
AM2	2.8 ± 1.2	3.0 ± 1.6	3.2 ± 2.2	5.3 ± 1.3	5.8 ± 1.6	6.2 ± 1.7
AM3	2.8 ± 1.4	2.7 ± 1.3	3.1 ± 2.0	4.9 ± 1.1	5.4 ± 0.6	6.3 ± 1.2

Condition coding: slow speed (V1), self-selected speed (V2), fast speed (V3), natural walking –no APO- (NW), transparent mode -APO shadows the wearer- (TM), low assistance (AM1), moderate assistance (AM2), high assistance (AM3)

For the pelvis cuff, pHR-ID, which ranges from 2.0 ± 0.6 mm in V2-TM to 3.1 ± 2.0 mm in V3-AM2, shows non-significant differences related to speed and significant differences associated with the walking modalities.

For the right cuff, the pHR-ID values, which range from 4.0 ± 0.6 mm in V1-TM to 6.3 ± 1.2 mm in V3-AM3, show significant differences related to both speed and assistance increments.

### Human-robot kinematics discrepancy

**Fig. 8:**
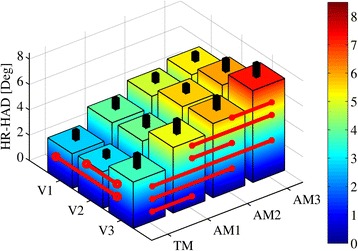
Hip kinematics and kinetics measured by the motion capture system (MCS, *black dashed lines*) and by the APO encoders (APO, *light green dashed lines*) for all speeds (slow (V1, first column), self-selected (V2, second column), fast (V3, third column)) and walking conditions (transparent mode -APO shadows the wearer- (TM, *blue lines*, first row), low assistance (AM1, *black lines*, second row), moderate assistance (AM2, *red lines*, third row), high assistance (AM3, *dark green lines*, forth row)). Each line represents the stride average and one SD band for each trial from one representative subject

**Fig. 9:**
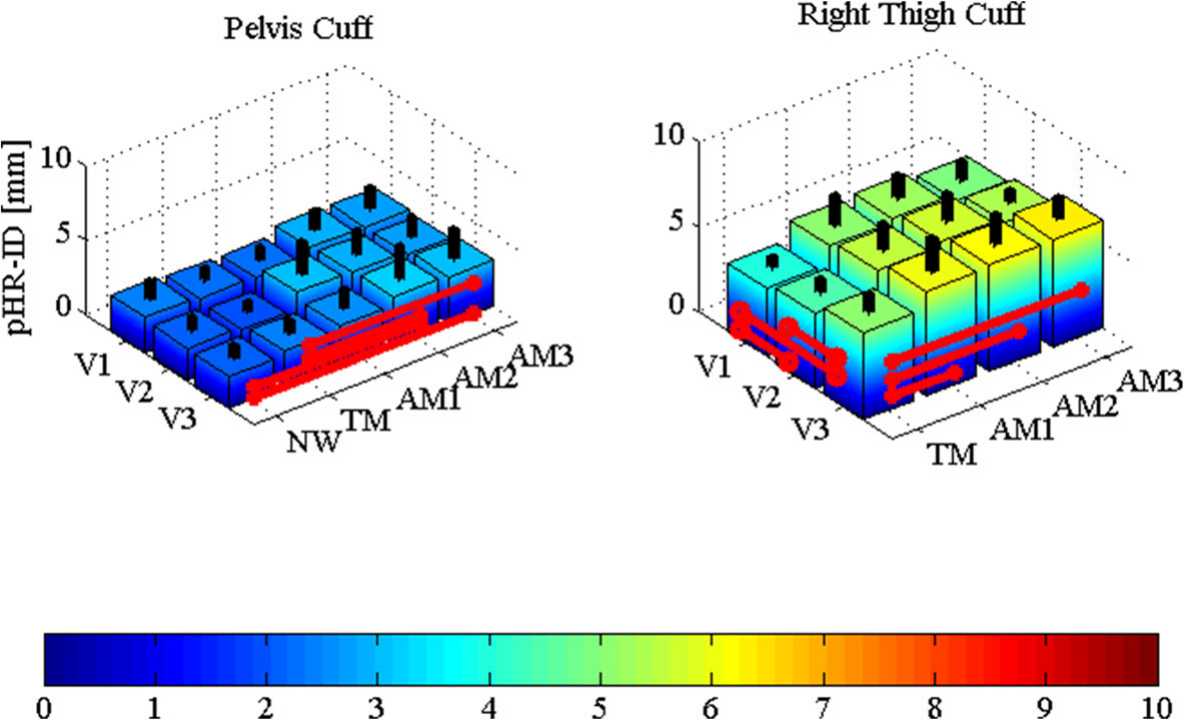
Mean ± SD of human-robot hip angle deviation (HR-HAD) for all speeds (slow (V1), self-selected (V2), fast (V3)) and walking conditions (transparent mode -APO shadows the wearer- (TM), low assistance (AM1), moderate assistance (AM2), high assistance (AM3)). *Black columns* represent one SD band. *Red lines* show significant (a two-way ANOVA (*p* < 0.05) with Fisher LSD post-hoc) differences

In Fig. 8, we show the human and APO kinematics at the hip f/e joint for one representative subject. We report a positive trend in the difference between the two trajectories that is associated both with speed and assistance increment. This difference arises between 40 and 90% of the stride cycle (torque delivery intervals from all the subjects are included in this range), when the flexion torque is delivered. When comparing walking modalities, all differences in HR-HAD are significant; when comparing speeds, the differences between V3 and V1/V2 are significant. HR-HAD ranges from 2.7 ± 0.8° in V1-TM to 7.2 ± 1.1° in V3-AM3 (see Fig. 9 and Table 6).

**Table 6:** Mean ± SD of HR-HAD categorized by speed and walking modality

Condition	V1 [Deg]	V2 [Deg]	V3 [Deg]
TM	2.7 ± 0.8	2.9 ± 0.6	3.6 ± 1.2
AM1	4.0 ± 0.9	4.1 ± 0.9	5.2 ± 1.1
AM2	4.9 ± 0.7	5.6 ± 0.7	5.9 ± 1.0
AM3	5.3 ± 0.7	6.1 ± 1.0	7.2 ± 1.1

Condition coding: slow speed (V1), self-selected speed (V2), fast speed (V3), transparent mode -APO shadows the wearer- (TM), low assistance (AM1), moderate assistance (AM2), high assistance (AM3)

## Discussion

### Spatio-temporal parameters

From the analysis of the spatio-temporal parameters, we deduce that speed significantly influences cadence and stance time. These results are expected and consistent with normative data [36]. The effect of wearing the APO in the TM and AMs on the cadence and step length is not significant, while the effect on the stance time is significant but not relevant (~1% of stride cycle variation). Hence, from the point of view of the spatio-temporal parameters, the APO does not affect physiological walking either in the TM or in the AMs.

### Kinematics

When considering the ROM, the effect of speed is expected and consistent with normative data. The effect of assistance both on the hip and knee f/e is significant, but the increased hip flexion is compensated by an increased knee flexion, which maintains the step length stable. This is most probably due to the natural dynamic coupling between the thigh and shank, which induces an increased f/e angle peak at the knee in response to the torque delivered at thigh level. This result is in line with the study reported in [31], in which the assistive controller used in this work was validated on the treadmill-based LOPES platform. This compensation mechanism seems not to involve ankle f/e.

While the ROM is an excellent indicator of the overall joint kinematics, it only accounts for a peak-to-peak difference over the stride. For this reason, it is interesting to consider parameters with higher content of information regarding the deviation from natural kinematics over the stride period. H-HAD, H-KAD, and H-AAD take into consideration a point-by-point difference of the joint angle trajectories between NW and other walking modalities during each interval of the stride cycle. A major deviation from natural kinematics would be a symptom of a hindering effect of the APO on the human joint. Nevertheless, these indicators are of the same order of magnitude as the average intra-subject variability (see Table 3 and Table 4). The deviation from natural kinematics introduced by wearing the exoskeleton in the TM/AM can be considered comparable with physiological kinematics variability and, hence, negligible. Therefore, the APO hinders physiological joint kinematics minimally. Furthermore, the deviation from NW kinematics is smaller in the TM than in the AMs for hip and ankle joints, while the differences are not significant for the knee joint (see Fig. 5). This proves that wearing the exoskeleton in the TM produces the lowest level of deviation from physiological walking among walking modalities.

It is worth mentioning that the registered average pelvis anteversion of 10 ± 1° caused by wearing the APO is consistent with previous studies, but additional research should be done in order to assess if such a deviation may cause noxious effects in terms of injury or discomfort in young adults carrying a light-weighted backpack (8 kg). Previous research has indicated an increased lumbosacral force in the case of 15%–20% of body weight carriage [37]; however, in this study, the APO represents in average 10% of the body weight of the participants.

### Physical human-robot interface displacements

Displacements between the cuffs and corresponding body segments are indicators of possible joint rotation axis misalignments and may produce pressure sores during exoskeleton usage. Absence of relative displacements is hardly obtainable owing to skin elasticity and compliance of soft tissue, although they must be kept as low as possible. The results show the pelvis cuff to be the most stable among all the cuffs in terms of pHR-ID. Indeed, the averaged pHR-ID value across speeds during NW is 2.2 ± 0.2 mm, while in the case of assistance, it takes its highest value of 3.2 ± 2.2 mm, only 1 mm higher. For the right thigh cuff, pHR-ID is in general two times higher than that of the pelvis cuff but still not critical in terms of pain or risk of injury for the user. In fact, in [38], the authors reported no skin damage or pain for skin strains of up to 11.7 mm on the forearm. The difference between the thigh and pelvis pHR-IDs is mainly due to the following. (i) The presence of large active muscles that can cause shifts of both cuffs and thigh markers. (ii) The different geometrical properties of the cuffs; the quasi-cylindrical shape of the thigh cuffs facilitates parallel and rotational shifts along the femoral axis. Instead, the pelvis cuff is molded to firmly lean against the iliac crests of the wearer, preventing downward shifts. Its geometry also impedes rotational shifts around the vertical axis. (iii) The inertia of moving masses, which increases more consistently at the thigh level, particularly at higher speeds, i.e., for larger accelerations. Since pHR-ID is below 1 cm, we can conclude that the APO is very unlikely to cause discomfort or injury due to excessive displacement between the cuff and body parts under normal use (i.e., when the peak torque—normalized to bodyweight—remains below 0.14 Nm/kg). This is confirmed by the fact that no subject reported pain or discomfort during usage.

One of the main limitations of this study is the lack of a direct measure of JAxM between the human and the robotic hip joints. Nevertheless, HR-HAD together with pHR-ID may provide important information regarding the appearance of possible injuries when using an exoskeleton. In the case or rigid WRs, the presence of large or small HR-HAD and pHR-ID can only imply large or small JAxM, respectively, since the human-robot kinematic configuration is uniquely determined by the human-robot kinematic discrepancy and the body-cuff relative displacements. The exoskeleton presented in this study has a certain degree of mechanical compliance owing to: (i) the finite stiffness of the structure, (ii) mechanical backlash, and (iii) the integration of pDoF mechanisms; the former two items are negligible with respect to the latter one. Despite compliance, when pHR-ID and HR-HAD are large, they can be considered as indicators of major JAxM, which may produce pain or discomfort, since JAxM is the only plausible cause of large pHR-ID and HR-HAD. Instead, when small HR-HAD and pHR-ID are measured, they cannot provide information regarding major or minor JAxM because the compliance of pDoF may absorb the parasitic forces induced by JAxM. Nevertheless, the evaluation of pHR-ID and HR-HAD may provide insight regarding eventual pain or discomfort to the user. Indeed, if natural kinematics is not altered by the exoskeleton (H-HAD, H-KAD, H-AAD) and no pain or discomfort is reported by the wearer at the physical interface (low pHR-ID) and articulations, possible JAxM may be considered not critical for the exoskeleton ergonomics in terms of risk of injury.

The presented results show a significant increment in the human-robot kinematics discrepancy associated with both speed and assistance level increase. When wearing the APO in the TM, the HR-HAD is the lowest; then, it increases with the increment of delivered assistance (namely, with the increment of the proportional gain K_v_), mostly between 40% and 90% of the gait cycle. It is worth noting that since the assistive strategy relies on the APO kinematics to determine the delivered torque profile, the larger the HR-HAD, the worse the match of the delivered torque envelope with the articulation biomechanics. Furthermore, the data show that HR-HAD is significantly dependent on the level of assistance (*K*_*v*_). Thereby, exceeding a body-weight-normalized torque limit of 0.14 Nm/kg would result in both a suboptimal human-robot kinematic coupling and a torque profile that is not compliant with human biomechanics, thus perceived as uncomfortable by the wearer. Hence, the measured HR-HAD seems to be not critical in terms of comfort and ergonomics, considering that the average peak torque—normalized for bodyweight—is below 0.14 Nm/kg.

Nevertheless, possible causes of HR-HAD are: (i) compression of soft tissues at the thigh and pelvis level and shifts of the orthotic shells during torque delivery (namely, pHR-ID), (ii) compliance of the APO mechanical structure (pDoFs), and (iii) misalignment between human and APO hip joint rotation axes (JAxM). Regarding the first cause, we observe that body-relative cuff shifts coincide with pHR-ID, which is composed of two components: one perpendicular to the interaction surface (e.g., compression of soft tissues) and one tangential (e.g., skin stretching and friction). Even in the worst case, in which we assume pHR-ID to be equal to one of the two components (e.g., only the tangential), the effect on comfort and risk of injury is negligible [38], regardless of direction. The other two causes are closely related since, in the case of JAxM, the parasitic load on the hip articulation may be absorbed by the chain of pDoFs, inducing a change in the APO kinematic configuration, which will be reflected by an increased HR-HAD.

Since physiological kinematics is only minimally perturbed by the APO (low H-HAD, H-KAD, H-AAD) and no subject reported pain during the execution of all trials, as confirmed by the low pHR-ID, we may consider the APO ergonomic, regardless of actual JAxM.

## Conclusions

In this paper, we explored the problem of physical human-robot interaction with a WR designed according to ergonomic criteria [7, 10, 11]. Furthermore, we proposed a metric to evaluate the quality of the interaction through ergonomics-related indicators such as: (i) deviation from natural kinematics and spatio-temporal parameters, (ii) human-robot kinematic discrepancy, and (iii) physical human-robot interface displacements.

The analysis of human kinematics and spatio-temporal parameters provides a global framework to investigate the impact of wearing the exoskeleton in the TM and AMs on physiological walking. In the case of healthy subjects, minimal deviation from natural walking is expected. As several parameters (e.g., spatio-temporal, ROM, average angle profiles) must be taken into account during the kinematics evaluation, we believe that the proposed indicators—H-HAD, H-KAD, and H-AAD—may provide a concise description of the deviation from natural kinematics, reducing the number and complexity of parameters to be considered.

The analysis of the human-robot kinematics discrepancy and physical human-robot interface displacements aims at performing an in-depth investigation of the ergonomics of physical human-robot interaction. Large HR-HAD and pHR-ID values are indicators of possible JAxM and instability at the physical human-robot interface. Hence, they can explain the deviations from natural kinematics (if any) and guide engineers towards improved mechanical design.

The APO mechanics and actuation cause no relevant interference in human locomotion. Indeed, human kinematics was not affected by the APO under all conditions that we tested. In addition, the physical human-robot kinematic coupling is reliable. Hence, there was no relevant relative displacement between the orthotic cuffs and corresponding anatomical segments under all tested conditions. These facts prove that the adopted mechanical design for passive DoFs allows an effective human-robot kinematic coupling.

The proposed methodologies and indicators may also be useful for the assessment of other research and commercial platforms. Nevertheless, additional work must be done to define a more quantitative scale for the evaluation of ergonomics, where each parameter is grounded on the evaluation of ultimate determinants (such as pain, discomfort, and risk of injury). In particular, a significant improvement would be to directly measure JAxM and derive its relation with pHR-ID and HR-HAD and the consequent discomfort or pain experienced by the user.

Nevertheless, we believe that the proposed metrics could represent a valid tool to obtain a quantitative measure of the ergonomics of a WR according to a theoretical framework in which the risk of injury is caused by JAxM, pHR-ID, and HR-HAD. In the future, their precise relationship must be analyzed in extensive studies relating each parameter to the eventual occurrence of injuries or perception of discomfort after regular long-term use.

## Abbreviations

a/a: Abduction/adduction
AM: Assistive mode
APO: Active pelvis orthosis
f/e: Flexion/extension
H-AAD: Human ankle angle deviation
H-HAD: Human hip angle deviation
H-KAD: Human knee angle deviation
HR-HAD: Human-robot hip angle deviation
i/e: Internal/external rotation
JAxM: Joint axes misalignment
MCS: Motion capture system
NW: Natural walking
pDoF: Passive degrees of freedom
pHR-ID: Physical human-robot interface displacement
RMS: Root mean square
ROM: Range of motion
SD: Standard deviation
TM: Transparent mode
V: Velocity
WR: Wearable robot
