# Erratum to: Physical human-robot interaction of an active pelvis orthosis: toward ergonomic assessment of wearable robots

**DOI:** 10.1186/s12984-017-0262-x

**Published:** 2017-06-05

**Authors:** Nicolò d’Elia, Federica Vanetti, Marco Cempini, Guido Pasquini, Andrea Parri, Marco Rabuffetti, Maurizio Ferrarin, Raffaele Molino Lova, Nicola Vitiello

**Affiliations:** 1 The BioRobotics Institute, Scuola Superiore Sant’Anna, viale Rinaldo Piaggio, 34, 56025 Pontedera, Pisa, Italy; 2 Fondazione Don Carlo Gnocchi IRCCS, Florence, Italy; 3 Fondazione Don Carlo Gnocchi IRCCS, Milan, Italy

## Erratum

The original article [1] contained errors whereby Figure citations were incorrectly numbered and cited within the text.

The original article has now been updated to reflect the correct Figure numbers and their respective citations within the text.

## References

[1] d’Elia N (2017). Physical human-robot interaction of an active pelvis orthosis: toward ergonomic assessment of wearable robots. J Neuroeng Rehabil.

